# Young Athletes Perceiving Greater Improvement After Return to Sport Bridge Program Sustained More Ipsilateral ACL Graft or Contralateral ACL Injuries During Their First Season Back: An Observational Study

**DOI:** 10.3390/jfmk10030335

**Published:** 2025-08-30

**Authors:** John Nyland, Brandon Pyle, Samuel Carter, Ryan Krupp, David N. M. Caborn

**Affiliations:** 1 Norton Orthopedic Institute, Louisville, KY 40241, USA; 2 Department of Orthopaedic Surgery, University of Louisville, Louisville, KY 40202, USA

**Keywords:** patient reported outcome measures, anterior cruciate ligament, return to sports, adolescent sports

## Abstract

**Objective:** Anterior cruciate ligament (ACL) graft failure or contralateral ACL injury after returning to sport (RTS) post-ACL reconstruction remains problematic. Re-injury prevention programs that “bridge” standard physical therapy and release to unrestricted sports participation can help. This observational study evaluated the characteristics of athletes who sustained an ipsilateral ACL graft or contralateral ACL injury after RTS bridge program participation. **Materials and Methods:** Comparisons were made between RTS bridge program participants who either had or had not sustained an ipsilateral ACL graft or contralateral ACL injury following RTS. Post-program objective physical function tests, pre- and post-program Knee Outcome Survey Sports Activity Scale (KOS-SAS), global sports activities knee function scores, sports activities knee function rating improvements, and post-program sport performance ability perceptions were evaluated. **Results:** A total of 204 athletes (19.7 ± 6 years of age, 108 males) completed the RTS bridge program and were released back to sports at 8.5 ± 2.3 months post-surgery. Groups had similar pre-morbid performance level restoration perceptions. Taller and heavier male athletes displayed greater single leg triple hop for distance magnitude, and quicker single leg timed hop, single leg timed crossover hop, and NFL 5-10-5 and NFL “L” times. Bilateral physical function test symmetry results did not differ between groups. By 7.8 ± 4 years post-surgery, 17 subjects sustained either ipsilateral ACL graft injury (*n* = 6) or contralateral ACL injury (*n* = 11), with a similar frequency between males and females (*p* = 0.30). Athletes who sustained an ipsilateral ACL graft or contralateral ACL injury were younger, and more often scored ≥ 25th percentile for post-program global sports activities knee function and KOS-SAS scores; more frequently had two-level overall sports activities knee function rating improvements; and tended to sustain this new knee injury during the initial RTS season. **Conclusions:** Factors other than physical function or performance capability may possess a strong influence on ipsilateral ACL graft or contralateral ACL injury following RTS bridge program participation.

## 1. Introduction

Primary and secondary anterior cruciate ligament (ACL) injuries continue to increase in young athletes, with restoration of pre-morbid performance levels remaining evasive, and with high surgical knee re-injury or contralateral knee injury risk [1,2,3,4,5,6,7]. Reports have referred to young athlete age being the “proxy” for high ipsilateral ACL graft or contralateral ACL injury risk, particularly during single leg landing and pivoting movements [8,9]. The psychological profile of many youth athletes may also serve as both a “surrogate” to earlier return to sports (RTS) and increased ipsilateral ACL graft or contralateral ACL injury risk, often in the presence of poorly developed lower extremity neuromuscular control [10,11,12]. Psychological profile factors such as high confidence, low fear, poor cognitive appraisal capability, and high athletic identity, which tend to be embedded in adolescence, have been associated with increased ipsilateral ACL graft or contralateral ACL injury risk [13,14,15,16]. Interestingly, by young adulthood (approximately 25 years of age) these factors become less concerning and athletic ACL injury or re-injury risks decrease [4,11].

An initial study reported that RTS bridge program implementation following physical therapy enabled most subjects (84%, 126/150) to RTS at or above their perceived pre-injury skill/performance level post-ACL reconstruction [17]. A later study identified the strong influence of global sports activity knee function scores among adolescent athletes that sustained an ipsilateral ACL graft or contralateral ACL injury [18]. These study findings suggested that supplementing primary ACL reconstruction and physical therapy with a RTS bridge program [19] with weekly guided training sessions and homework improved bilateral lower extremity and core function through progressive intensity sport movements [19]. This led to improved patient outcomes and decreased ipsilateral ACL graft and contralateral ACL injury rates [17,18,19]. This observational study attempted to better delineate the characteristics of subjects who sustained either ipsilateral ACL graft or contralateral ACL injury after release to unrestricted sports participation following RTS bridge program completion [17,18,19].

## 2. Materials and Methods

Institutional Review Board Approval: This observational study was conducted in accordance with the Declaration of Helsinki and approved by the Research Ethics Committee of Spalding University (REC #02-192020, 19 February 2020). All subjects provided written informed consent, and all training or testing was performed on the sport playing surface with proper footwear.

Inclusion criteria: All subjects that were post-primary ACL reconstruction, had finished standard physical therapy, and had successfully completed the RTS bridge program [17] were included in this study.

Exclusion criteria: Subjects who did not complete the RTS bridge program, that had sustained injuries to other body regions, or who had undergone revision ACL reconstruction were not eligible for study participation.

Data collection: Demographic data was collected at RTS bridge program initiation. Subjective perceived knee function and performance data was collected at RTS bridge program initiation and at final testing. All objective physical function tests were performed at final testing prior to program completion. Most subjects had sustained an index ACL injury playing soccer, basketball, or American football. Reconstruction of the ACL had been performed primarily using a quadricep tendon autograft, bone-patellar tendon-bone autograft, hamstring tendon autograft, or a tibialis anterior tendon allograft, and graft fixation was achieved primarily using either suspensory buttons or interference screws [17]. No subjects underwent ACL repair. The percentage of subjects who underwent concomitant surgical procedures including partial medial meniscectomy (15%), partial lateral meniscectomy (5%), medial meniscus repair (15%), or lateral meniscus repair (3%), or that had a grade I or II medial (20%) or lateral (4%) collateral ligament sprain (treated non-operatively) was similar between groups [17]. All subjects had complied with post-surgical physical therapy and RTS bridge program attendance. At RTS, no subject displayed significant knee joint laxity (≤2 mm) based on a manual maximum Lachman test performed by the primary investigator (8.5 ± 2.4 months post-surgery). At program completion, all subjects could perform 8-repetition maximum single leg presses with ≥ bodyweight resistance bilaterally. In addition to demographic variables, groups that had or had not sustained an ipsilateral ACL graft or contralateral ACL injury following RTS were compared for objective physical function test performance at program completion, including post-surgical and non-surgical lower extremity performance during 20 m single leg timed hop, 20 m single leg timed crossover hop, single leg triple hop for distance, NFL ‘L’, and NFL ‘5-10-5’ tests [17,18,19,20]. Groups were also compared for pre-program Knee Outcome Survey Sports Activity Scale (KOS-SAS), global sports activities knee function scores, and sports activities knee function rating improvements which were re-evaluated at program completion; post-program KOS-SAS scores, global sports activity knee function scores, sports activities knee function rating improvements, and post-program sport performance ability perceptions (worse versus same or better) were also evaluated. The global sports activities knee function score question was, “How would you rate the current function of your knee during sports activities on a scale from 0–100 with 100 being your level of knee function prior to your injury and 0 being the inability to perform any sports activities?”. Overall sports activities knee function rating options were normal, nearly normal, abnormal, and severely abnormal.

### Statistical Analysis

Post-program objective physical function tests, KOS-SAS, global sport activity knee function scores, and overall sport activity knee function ratings were compared between groups that either had or had not sustained an ipsilateral ACL graft or contralateral ACL injury. Shapiro–Wilk tests were used to evaluate data normality. Most outcome variables displayed normal distributions; therefore, comparisons were made using two-way ANOVA (re-injury group, gender). However, global sport activity knee function and KOS-SAS scores did not display normality; therefore, non-parametric Mann–Whitney ‘U’ tests were used to compare these variables. Chi-square tests were used to compare categorical group data. An alpha level of *p* ≤ 0.05 was selected to indicate statistically significant differences. All statistical procedures were performed using specialized software (IBM-SPSS version 29.0, Chicago, IL, USA).

## 3. Results

After completing the RTS bridge program, 204 athletes (19.7 ± 6 years of age, 108 males) were released at 8.5 ± 2.4 months post-surgery. Groups had similar perceptions of RTS at the same or better pre-morbid sport performance level (Table 1).

Athletes who sustained ipsilateral ACL graft or contralateral ACL injury more frequently scored ≥ 25th percentile for the post-program KOS-SAS score (94.1%, 16/17 vs. 77%, 144/187) (*p* = 0.04) and for the post-program global sports activities knee function score (94.1%,16/17 vs. 77.5%, 145/187) (*p* = 0.009). Athletes who sustained ipsilateral ACL graft or contralateral ACL injury also had more subjects that perceived a two-level sports activities knee function rating improvement 14/17 (82.4%) than the group that did not 93/187 (49.7%) (*p* = 0.01). Objective physical function test limb symmetry results did not differ between athletes who had, or who had not, sustained ipsilateral ACL graft injury or contralateral ACL injury. Male athletes were taller and weighed more than females displaying greater single leg triple hop for distance magnitude, and quicker single leg timed hop, single leg timed crossover hop, and NFL 5-10-5 and NFL “L” times; however, symmetry was comparable (Table 2).

By 7.8 ± 4 years post-surgery, 17 subjects had sustained either ipsilateral ACL graft (*n* = 6) or contralateral ACL injury (*n* = 11) with a similar frequency between males and females (*p* = 0.30). Six injuries (35%) occurred from contact to the surgical knee (*n* = 3, each during American football; two cases had used BPTB autografts and one had used a quadriceps tendon autograft) or to the contralateral knee (*n* = 3, one American football, one wrestling, and one soccer). Eleven injuries (65%) involved non- or in-direct contact to the surgical knee (*n* = 3, two American football, one soccer; two cases had used a quadriceps tendon autograft and one had used a BPTB autograft) or to the contralateral knee (*n* = 8, three soccer, two basketball, two lacrosse, and one taekwondo).

Post-program ipsilateral ACL graft or contralateral ACL injuries occurred during the initial RTS season (47%, *n* = 8), 1 year after RTS (*n* = 5, 29%), 2 years after RTS (*n* = 2, 12%), 5 years after RTS (6%, *n* = 1), and 10 years after RTS (6%, *n* = 1). Athletes who sustained these injuries were younger at RTS bridge program initiation (17.1 ± 1.8 years vs. 20.9 ± 9 years, *p* = 0.01) and most ipsilateral ACL graft or contralateral ACL injuries occurred during the initial RTS season (8/17, *p* = 0.01).

## 4. Discussion

The most important study finding was that, compared to athletes who had not sustained ipsilateral ACL graft or contralateral ACL injury, athletes that sustained injury had the following characteristics: 1. Were younger; 2. more often scored ≥ the 25th percentile for post-program global sports activities knee function and KOS-SAS scores; 3. more frequently displayed a two-level (from severely abnormal to nearly normal, or abnormal to normal) overall sports activities knee function rating improvement; and 4. more often sustained ipsilateral ACL graft or contralateral ACL injury during the initial RTS season. Overall, these factors related more to ipsilateral ACL graft or contralateral ACL injury risk than any objective physical function test.

Following physical therapy and RTS bridge program participation, the current study found males and females to have similar ipsilateral ACL graft or contralateral ACL injury risk. Identification of more frequent two-level overall sports activities knee function rating improvement suggests that young athletes who sustained an ipsilateral ACL graft or contralateral ACL injury may have perceived themselves to possess greater sports knee function at RTS bridge program completion than their peers who did not sustain ipsilateral ACL graft or contralateral ACL injury. Lastly, 8 of the 17 subjects who sustained ipsilateral ACL graft or contralateral ACL injury during the initial RTS season experienced it during games (*n* = 6, 3 non- or in-direct contact, and 3 contact mechanism) rather than practices (*n* = 2, 1 non- or in-direct contact, and 1 contact), and 5 of the 8 (62.5%) ipsilateral ACL graft or contralateral ACL injuries that occurred during the initial RTS season were sustained playing American football. Interestingly, groups did not differ regarding their perception of whether or not they had RTS at the same or better performance level.

The fact that group differences were not observed for objective physical function tests, or for subjective perceived sport performance level, suggests that other factor(s) may have contributed to the higher perceived global sports activities knee function scores and sports activities knee function rating improvements observed for this group. Although not evaluated in the current study, diverse factors such as athletic identity [21,22], confidence or self-efficacy [23,24,25], kinesiophobia or fear [24,26,27], team role (starter, captain, leader, etc.) [28], health locus of control [29,30], cognitive appraisal [31] or perceived self, or peer, parent/guardian, or coach pressure [32], among other factors, may have contributed to the higher perceived sport knee function scores and ratings observed in this group.

An important study limitation is that the KOS-SAS focused exclusively on physical symptoms such as knee pain, grating/grinding, stiffness, swelling, slipping or giving way, buckling, or weakness, and physical function limitations such as running straight ahead, jumping and landing on the side of the involved knee, stopping and starting quickly, and cutting and pivoting on the injured leg [33]. Subject perceptions of fear, confidence, cognitive appraisal, emotions, and athletic identity were not assessed. Supplemental use of The Anterior Cruciate Ligament Return to Sport after Injury Survey (ACL-RSI) would have provided a responsive inventory of subject psychological profiles including confidence, re-injury thoughts, nervousness, frustration, fear, thoughts about having to go through surgery and rehabilitation again, and relaxation level while playing a sport [34,35,36]. Another important study limitation is that this retrospective analysis of prospectively collected data does not allow for direct cause and effect relationship determination. Since comparisons were made between pre-program KOS-SAS, global sports activities knee function score, and sports activities knee function rating improvement re-evaluations at program completion, recall bias may also have influenced study results. These data were collected in this manner to enable subjects to make direct comparisons between their perceived sports activities knee function post-physical therapy, prior to having participated in the 8-week-long program with their current sports activities knee function perceptions. Lastly, factors which also may have influenced continued ACL injury risk such as family history of ACL rupture, the presence of positive lower extremity Beighton signs or generalized joint laxity [37,38,39,40], or abnormal tibial surface/femoral notch morphology [41,42,43,44] were not evaluated in this study.

The mean age of the ipsilateral ACL graft or contralateral ACL injury group (17.1 ± 1.8 years) suggests that most were middle adolescent age [15]. Given the different ACL reconstruction grafts and fixation methods used, and sports played, these results may generalize to other populations of middle adolescent-aged athletes. During this developmental period the young athlete must adjust to a new physical sense of self, experiencing rapid and profound physical changes triggered by hormones acting on different body parts (Table 3) [45,46,47]. During middle adolescence, body appearance becomes more important with juxtaposed periods of excessive and low physical activity levels [45,46,47]. Grohman et al. [48] reported that accumulated microtrauma damage over a 6 month time period of pre-season sport preparation training may have contributed to sudden ACL rupture during the season. Appetite tends to increase during growth spurts and markedly decreases between them [45,46,47]. Additionally, during this time period, the athlete develops increased sleep needs, and experiences ongoing cognitive and behavioral development [45,46,47]. Each of these adjustments might influence native ACL recovery from training-related accumulated microtrauma [49,50].

In addition to physical symptoms and function [30], and psychological readiness assessments [36], the multiple bodily system changes that occur during middle adolescence suggests that decisions about RTS after ACL injury, surgery, rehabilitation, and transitional RTS bridge program participation would benefit from more comprehensive psychological-readiness information within the context of the adolescent athlete’s developmental phase and with input from important stakeholder or social support groups (parents/guardians, coaches, teachers, etc.) [49,50].

Although the current study did not directly measure subject psychological readiness factors at the time of RTS, the preponderance of more frequent post-program KOS-SAS scores and global sports activities knee function scores ≥ the 25th percentile and the more frequent two-level sports activities knee function rating improvements that were observed suggest that subjects who sustained ipsilateral ACL graft or contralateral ACL injury following RTS may have had greater confidence and less fear than the group that did not sustain ipsilateral ACL graft or contralateral ACL injury. With a mean age of 17.1 years [interquartile range = 2 years], middle adolescent-aged subjects that sustained ipsilateral ACL graft or contralateral ACL injury may also have possessed a stronger athletic identity than the group that did not [51,52]. Given the self-, peer, parent/guardian, and coach pressures that often influence high school age (14–18 years) athletes and the multiple bodily system development that occurs from early-to-late adolescence prior to transitioning into young adulthood (>24 years of age), it is important that primary or secondary ACL injury prevention programs better match specific developmental needs. Trying to interpret patient-reported outcome measures (PROM) in adolescent athletes is like chasing a “moving target” of rapidly changing behaviors and perceptions, which may reduce their reliability and validity [53].

Of the eight subjects who sustained ipsilateral ACL graft or contralateral ACL injury during the initial RTS season (≤1 year), most (*n* = 5) occurred in American football (four during games, and one during practice), two occurred in soccer games, and one occurred in a lacrosse game. Four involved direct contact (three males, one female) and four involved non- or in-direct contact mechanisms (three males, one female). Of these eight athletes, six also had leadership roles on their respective teams as American football quarterbacks (*n* = 2); key running backs (*n* = 3); or as a starting basketball point guard (*n* = 1). Team leadership roles may have been associated with many of these athletes possessing stronger athletic identities (social identity, exclusivity, or both) [54,55] than the group that did not sustain ipsilateral ACL graft or contralateral ACL injury. Athletic identity is associated with sport skills, confidence, and social interaction from sport [22], and how sport involvement serves as an important social statement about who the individual is as a person and how they want others to perceive them. Social identity is the athletic identity sub-component that measures an athlete’s personal connection to their “athlete role”, while exclusivity represents how much they identify exclusively in their sport role [55]. In the current time of greater and earlier sport specialization and “professionalized” youth sport training, the strength of these athletic identity subcomponents may be stronger and more frequently observed [56]. The higher global sports activities knee function scores and sports activities knee function rating improvements that were observed among athletes who sustained ipsilateral ACL graft or contralateral ACL injury following RTS suggest that they may have possessed stronger athletic identities which influenced their sports confidence and fear levels [27].

An associated factor with the greater team leadership roles for those athletes who sustained an ipsilateral ACL graft or contralateral ACL injury may also have been a greater likelihood for returning earlier to higher volume playing time following their RTS, particularly during key games. No matter how progressive the rehabilitation or RTS bridge program that an athlete participates in, it is unlikely to closely replicate the intensity, total training volume, and stresses of their chosen sport. For this reason, after being released to unrestricted RTS participation, each athlete had been instructed to complete two full weeks of practice without experiencing knee joint symptoms prior to playing in a game and was also prescribed individualized maintenance “neuromuscular control activity homework”. Follow-up with both the athletes and their coaches suggested that these guidelines were not always adhered to.

## 5. Conclusions

Athletes who sustained an ipsilateral ACL graft or contralateral ACL injury were younger, and more often scored ≥ the 25th percentile for post-program global sports activities knee function and KOS-SAS scores; more frequently displayed two-level overall sports activities knee function rating improvements; and tended to sustain the ipsilateral ACL graft or contralateral ACL injury during the initial RTS season. Although physical function milestones are important, following RTS bridge program participation, other factors may possess a greater influence on athletes who sustain an ipsilateral ACL graft or contralateral ACL injury. During middle adolescence, athletic identity is likely interwoven with high confidence, low fear, inadequate cognitive risk appraisal, greater self-pressure, and greater perceived pressure from peers, coaches, and parents/guardians.

## Figures and Tables

**Table 1:** Comparison of demographics and patient-reported outcome measures of the two groups. NA—not applicable.

	No ACL Graft or Contralateral ACL Injury (*n* = 187)		ACL Graft or Contralateral ACL Injury (*n* = 17)		Gender *p*	Group *p*	Interaction *p*
Gender (#)	97 male, 90 female		11 male, 6 female		NA	0.26	NA
	Male	Female	Male	Female			
Age (mean ± SD) years	20.9 ± 6	18.8 ± 6	17.8 ± 2.1	17.0 ± 2	0.33	0.01	0.70
Height (mean ± SD) (cm)	180.4 ± 8	169.5 ± 8	181.1 ± 5	163.4 ± 6	<0.001	0.16	0.08
Weight (mean ± SD) (kg)	85.6 ± 14	64.1 ± 8	84.4 ± 16	57.2 ± 8	<0.001	0.19	0.35
Body Mass Index (mean ± SD)	26.3 ± 4	22.3 ± 3	25.6 ± 4	21.3 ± 3	<0.001	0.38	0.89
Post-Surgery RTS (mean ± SD) months	8.1 ± 3	8.8 ± 2.5	8.0 ± 2	7.8 ± 1	0.52	0.64	0.08
Pre-Program KOS-SAS Score re-evaluation at program completion [median [IQR]	59.2 [22.6]	57.5 [31.5]	60.1 [40.6]	54.0 [52.4]	0.50	0.82	0.71
Post-Program KOS-SAS Score [median [IQR]	93 [8.9]	90.3 [5.9]	94.4 [5.1]	95.3 [4.3]	0.87	0.15	0.60
Post-Program KOS-SAS Score ≥ 25th percentile % (#)	76.3% (74/97)	77.9% (70/90)	100% (11/11)	83.3% (5/6)	0.34	0.04	NA
Pre-Program Global Sport Activities Knee Function Score re-evaluation at program completion [median [IQR]	55.8 [31.5]	55.2 [30]	50 [52.5]	47.4 [51.6]	0.80	0.29	0.88
Post-Program Global Sport Activities Knee Function Score [median [IQR]	91.3 [8]	91.0 [6.3]	94.5 [10]	94.6 [4]	0.97	0.16	0.94
Post-Program Global Sport Activities Knee Function Score ≥ 25th percentile % (#)	79.5% (77/97)	76.1% (68/90)	100% (11/11)	83.3% (5/6)	0.74	0.009	NA
Pre-Program Sports activities knee function rating (normal, nearly normal, abnormal, severely abnormal) % (#)	70% (78/97) (abnormal)	73.3% (66/90) (abnormal)	72.7% (8/11) (abnormal)	83.3 (5/6) (abnormal)	0.64	0.75	0.38
Post-Program Sports activities knee function rating (normal, nearly normal, abnormal, severely abnormal) % (#)	82.2% (80/97) (normal)	79.4% (71/90) (normal)	88.2% (9/11) (normal)	83.3%(5/6) (normal)	0.32	0.35	0.52
Two-level Sports activities knee function rating improvement from Pre- to Post-Program (normal, nearly normal, abnormal, severely abnormal) % (#)	54.6% (53/97) (abnormal to normal or severely abnormal to nearly normal)	44.9% (40/90) (abnormal to normal or severely abnormal to nearly normal)	90.9%, (10/11) (abnormal to normal or severely abnormal to nearly normal)	66.7% (4/6) abnormal to normal or severely abnormal to nearly normal)	0.12	0.009	NA
Same or Better Perceived Sports Performance Level Rating (same or better, or worse)	83/97 (85.9%)	75/90 (83.7%)	10/11 (90.9%)	5/6 (83.3%)	0.55	0.99	NA

**Table 2:** Comparison of the objective functional test performance of the two groups.

	No ACL Graft or Contralateral ACL Injury (*n* = 187)		ACL Graft or Contralateral ACL Injury (*n* = 17)		Gender *p*	Group *p*	Interaction *p*
	97 male, 90 female		11 male, 6 female				
Single Leg Triple Hop for Distance (Non-surgical) (mean ± SD) (cm)	486.4 ± 121	379.2 ± 73	525.3 ± 103	422.9 ± 39	0.001	0.19	0.94
Single Leg Triple Hop for Distance (Surgical) (mean ± SD) (cm)	471.3 ± 120	354.3 ± 67	507.7 ± 103	400.7 ± 33	<0.001	0.18	0.87
Single Leg Triple Hop for Distance Symmetry Index (mean ± SD) (%)	96.9 ± 5	93.7 ± 6	96.7 ± 5	94.8 ± 2	0.13	0.77	0.69
20 m Single Leg Timed Hop (Non-surgical) (mean ± SD) (s)	4.6 ± 0.8	5.9 ± 1.1	4.6 ± 0.8	5.1 ± 0.6	0.007	0.27	0.18
20 m Single Leg Timed Hop (Surgical) (mean ± SD) (s)	4.6 ± 0.8	6.2 ± 1.3	4.7 ± 0.9	5.1 ± 0.9	0.009	0.21	0.13
20 m Single Leg Timed Hop Symmetry Index (mean ± SD) (%)	99 ± 6	96 ± 6	99 ± 5	99.8 ± 0.4	0.60	0.42	0.40
20 m Single Leg Timed Crossover Hop (Non-surgical) (mean ± SD) (s)	5.6 ± 1.5	7.2 ± 1.7	5.3 ± 1.2	6.3 ± 1.2	0.015	0.28	0.63
20 m Single Leg Timed Crossover Hop (Surgical) (mean ± SD) (s)	5.6 ± 1.4	7.2 ± 1.7	5.3 ± 1.1	6.1 ± 1.0	0.028	0.19	0.47
20 m Single Leg Timed Crossover Hop Symmetry Index (mean ± SD) (%)	98.6 ± 7	99.6 ± 5	98.6 ± 5	103.6 ± 4	0.13	0.45	0.45
NFL “5-10-5” (Non-Surgical side first cut) (mean ± SD) (s)	4.9 ± 0.6	5.6 ± 0.7	4.9 ± 0.4	5.3 ± 0.6	0.005	0.42	0.32
NFL “5-10-5” (Surgical side first cut) (mean ± SD) (s)	4.8 ± 0.4	5.6 ± 0.7	4.9 ± 0.4	5.3 ± 0.6	0.001	0.46	0.23
NFL “5-10-5” Symmetry Index (%)(mean ± SD) (%)	99.8 ± 6	99.5 ± 3	100 ± 3	100 ± 3	0.91	0.76	0.96
NFL 3 Cone Shuttle “L” Drill (Non-surgical side cut) (mean ± SD) (s)	8.3 ± 0.9	9.3 ± 1.2	8.3 ± 0.6	9.2 ± 3	<0.001	0.56	0.50
NFL 3 Cone Shuttle “L” Drill (Surgical side cut) (mean ± SD) (s)	8.3 ± 0.8	9.3 ± 1.2	8.2 ± 0.5	9.3 ± 2	<0.001	0.65	0.49
NFL 3 Cone Shuttle “L” Drill Symmetry Index (mean ± SD) (%)	100 ± 3	100 ± 3	100 ± 2	100 ± 2	0.74	0.65	0.89

**Table 3:** Important developmental occurrences during early-to-late adolescence.

Early Adolescence (10–13 Years)		Middle Adolescence (14–17 Years)	Late Adolescence (≥18 Years)
Puberty starts with physical growth and increased sexual interest; **Females often have their peak growth spurt** Tanner signs appear; body changes cause curiosity, anxiety; Cognitive development with concrete, all-or-nothing thinking. Limited abstract thought capacity; **Self-conscious regarding appearance, peer judgment apprehension** Expanding intellectual interests, develop deeper moral thinking; Increased privacy needs; Explores greater independence from family, pushing boundaries, reacting to enforced limits		Physical growth continues; **males often have peak growth spurt** and voice lowering; Slowed physical growth for females, most develop regular menstrual periods; Interest in sexual relationships and identity increases; Striving for greater independence increases arguments with parents; More time spent with friends, less time spent with family; **Peer pressure peaks, becomes more self-involved, appearance is very important;** **Brain development continues with greater abstract thought, but emotions often drive decisions with actions based on impulses;** Begin to establish long-term goals and become interested in the meaning of life and moral reasoning	**Greater brain and cognitive development, less physical development;** **Increasing rational thought with developing self control; can delay gratification, and future planning;** **Increased individuality, and personal value development;** Greater independence, emotional stability, friendship and relationship stability; May begin to look at parents less as authority figures and more as peers.
**Potential Sports Orthopedic Considerations**			
1.	Family History of ACL Injury—If non- or in-direct contact, consider selection of a lower knee injury risk sport.		
2.	If positive knee Beighton sign [37,38,39,40]—Selection of a lower knee injury risk sport.		
3.	If positive abnormal tibial surface or femoral notch morphology [41,42,43,44]—Selection of a lower knee injury risk sport.		
4.	Confidence/Self-Efficacy [23,24,25]—Consider in relation to existing skill level and adolescence phase.		
5.	Fear/Kinesiophobia/Anxiety [24,25,26,27]—Consider in relation to existing skill level, injury history, and adolescence phase.		
6.	Athletic Identity [21,22,51,52]—Consider in relation to adolescence phase, desire for sport specialization or elite level training, and whether activity diversification is needed.		
7.	Primary Sport Selection(s)—Consider in relation to #1–3; if “+” consider selection of a lower knee injury risk sport.		
8.	Dedicated Sport Seasonality (in season, out of season (recovery) time periods)—Consider in relation to #1–3.		
9.	Injury/Re-Injury Cognitive Risk Appraisal [25,35]—Consider in relation to #1–3 and adolescence phase.		
10.	Social Support—Consider based on each adolescence phase and existing resources (parents/guardians, pediatrician, team physicians, coaches, and other stakeholders).		

## Data Availability

The raw data supporting the conclusions of this article will be made available by the authors on request.

## Abbreviations

The following abbreviations are used in this manuscript:

ACL	Anterior cruciate ligament
cm	Centimeter
IQR	Inter-quartile range
kg	kilogram
KOS-SAS	Knee outcome survey—sports activity scale
NFL	National Football League
PROM	Patient reported outcome measurement
RTS	Return to sports
SD	Standard deviation
s	Seconds

## References

[1] Ardern C.L., Taylor N.F., Feller J.A., Webster K.E. (2014). Fifty-five per cent return to competitive sport following anterior cruciate ligament reconstruction surgery: An updated systematic review and meta-analysis including aspects of physical functioning and contextual factors. Br. J. Sports Med..

[2] Webster K.E., Feller J.A., Whitehead T.S., Myer G.D., Merory P.B. (2017). Return to Sport in the Younger Patient With Anterior Cruciate Ligament Reconstruction. Orthop. J. Sports Med..

[3] Niederer D., Keller M., Wießmeier M., Vogt L., Stöhr A., Schüttler K.-F., Schoepp C., Petersen W., Pinggera L., Mengis N. (2024). The End of the Formal Rehabilitation Is Not the End of Rehabilitation: Knee Function Deficits Remain After Anterior Cruciate Ligament Reconstruction. J. Sport Rehabil..

[4] Wiggins A.J., Grandhi R.K., Schneider D.K., Stanfield D., Webster K.E., Myer G.D. (2016). Risk of Secondary Injury in Younger Athletes After Anterior Cruciate Ligament Reconstruction: A Systematic Review and Meta-analysis. Am. J. Sports Med..

[5] Webster K.E., Hewett T.E. (2019). What is the Evidence for and Validity of Return-to-Sport Testing after Anterior Cruciate Ligament Reconstruction Surgery? A Systematic Review and Meta-Analysis. Sports Med..

[6] Paterno M.V., Rauh M.J., Schmitt L.C., Ford K.R., Hewett T.E. (2014). Incidence of Second ACL Injuries 2 Years After Primary ACL Reconstruction and Return to Sport. Am. J. Sports Med..

[7] Paterno M.V., Rauh M.J., Schmitt L.C., Ford K.R., Hewett T.E. (2012). Incidence of contralateral and ipsilateral anterior cruciate ligament (ACL) injury after primary ACL reconstruction and return to sport. Clin. J. Sport Med..

[8] Webster K.E. (2021). Editorial Commentary: Anterior Cruciate Ligament Suture Repair Could Have High Failure Rates in Active Athletes of All Ages. Arthroscopy.

[9] Gokeler A., Dingenen B., Hewett T.E. (2022). Rehabilitation and Return to Sport Testing After Anterior Cruciate Ligament Reconstruction: Where Are We in 2022?. Arthrosc. Sports Med. Rehabil..

[10] Zarzycki R., Cummer K., Arhos E., Failla M., Capin J.J., Smith A.H., Snyder-Mackler L. (2024). Female Athletes With Better Psychological Readiness Are at Higher Risk for Second ACL Injury After Primary ACL Reconstruction. Sports Health.

[11] Edwards P.K., Ebert J.R., Joss B., Ackland T., Annear P., Buelow J.-U., Hewitt B. (2018). Patient Characteristics and Predictors of Return to Sport at 12 Months After Anterior Cruciate Ligament Reconstruction: The Importance of Patient Age and Postoperative Rehabilitation. Orthop. J. Sports Med..

[12] Butcher A.J., Ward S., Clissold T., Richards J., Hébert-Losier K. (2024). Maturation and biomechanical risk factors associated with anterior cruciate ligament injury: Is there a link? A systematic review. Phys. Ther. Sport.

[13] Piussi R., Beischer S., Thomeé R., Thomeé C., Sansone M., Samuelsson K., Senorski E.H. (2022). Greater Psychological Readiness to Return to Sport, as Well as Greater Present and Future Knee-Related Self-Efficacy, Can Increase the Risk for an Anterior Cruciate Ligament Re-Rupture: A Matched Cohort Study. Arthroscopy.

[14] Sawyer S.M., Azzopardi P.S., Wickremarathne D., Patton G.C. (2018). The age of adolescence. Lancet Child Adolesc. Health.

[15] Ueda Y., Matsushita T., Shibata Y., Takiguchi K., Ono K., Kida A., Nishida K., Nagai K., Hoshino Y., Matsumoto T. (2024). Psychological Readiness to Return to Sports at 3 Months Postoperatively and Risk of Second ACL Injury. Orthop. J. Sports Med..

[16] Hardin A.P., Hackell J.M. (2017). Committee on Practice and Ambulatory Medicine. Age Limit Pediatr..

[17] Nyland J., Greene J., Carter S., Brey J., Krupp R., Caborn D. (2020). Return to sports bridge program improves outcomes, decreases ipsilateral knee re-injury and contralateral knee injury rates post-ACL reconstruction. Knee Surg. Sports Traumatol. Arthrosc..

[18] Nyland J., Tomberlin C., Brey J., Carter S. (2024). Global knee function rating more strongly influences adolescent athletes that sustain a sports-related surgical ACL re-injury or contralateral ACL injury. Knee Surg. Sports Traumatol. Arthrosc..

[19] Nyland J., Pyle B., Richards J., Yoshida K., Brey J., Carter S. (2023). A clinical practice review of therapeutic movement-based anterior cruciate ligament reconstruction return to sports bridge program: The biological, biomechanical and behavioral rationale. Ann. Jt..

[20] Keller R.A., Mehran N., Austin W., Marshall N.E., Bastin K., Moutzouros V. (2015). Athletic performance at the NFL scouting combine after anterior cruciate ligament reconstruction. Am. J. Sports Med..

[21] McGinley J., Stapleton E., Gale E., Worrall H., Podvin C., Ellis H.B., Wilson P.L., Ulman S. (2024). Differences in athletic identity, sport participation, and psychosocial factors following anterior cruciate ligament rehabilitation in youth athletes. Front. Psychol..

[22] Edison B.R., Christino M.A., Rizzone K.H. (2021). Athletic Identity in Youth Athletes: A Systematic Review of the Literature. Int. J. Environ. Res. Public Health.

[23] Paterno M.V., Thomas S., VanEtten K.T., Schmitt L.C. (2022). Confidence, ability to meet return to sport criteria, and second ACL injury risk associations after ACL-reconstruction. J. Orthop. Res..

[24] Bullock G.S., Sell T.C., Zarega R., Reiter C., King V., Wrona H., Mills N., Ganderton C., Duhig S., Räisäsen A. (2022). Kinesiophobia, Knee Self-Efficacy, and Fear Avoidance Beliefs in People with ACL Injury: A Systematic Review and Meta-Analysis. Sports Med..

[25] Webster K.E., Feller J.A. (2018). Development and Validation of a Short Version of the Anterior Cruciate Ligament Return to Sport After Injury (ACL-RSI) Scale. Orthop. J. Sports Med..

[26] Chmielewski T.L., George S.Z. (2019). Fear avoidance and self-efficacy at 4 weeks after ACL reconstruction are associated with early impairment resolution and readiness for advanced rehabilitation. Knee Surg. Sports Traumatol. Arthrosc..

[27] Ferman B., Nyland J., Richards J., Krupp R. (2024). Adolescent Athletes with Stronger Athletic Identity Perceptions Have Weaker Fear Avoidance Perceptions During Musculoskeletal Injury Rehabilitation Return to Sports Preparation. J. Pediatr. Orthop..

[28] Podlog L., Wadey R., Stark A., Lochbaum M., Hannon J., Newton M. (2013). An adolescent perspective on injury recovery and the return to sport. Psychol. Sport Exerc..

[29] Nyland J., Cottrell B., Harreld K., Caborn D.N. (2006). Self-reported outcomes after anterior cruciate ligament reconstruction: An internal health locus of control score comparison. Arthroscopy.

[30] Nyland J., Mauser N., Caborn D.N. (2013). Sports involvement following ACL reconstruction is related to lower extremity neuromuscular adaptations, subjective knee function and health locus of control. Knee Surg. Sports Traumatol. Arthrosc..

[31] Baez S.E., Hoch M.C., Hoch J.M. (2020). Psychological factors are associated with return to pre-injury levels of sport and physical activity after ACL reconstruction. Knee Surg. Sports Traumatol. Arthrosc..

[32] Sheehan N., Summersby R., Bleakley C., Caulfield B., Matthews M., Klempel N., Holden S. (2024). Adolescents’ experience with sports-related pain and injury: A systematic review of qualitative research. Phys. Ther. Sport.

[33] Pantano K.J., Irrgang J.J., Burdett R., Delitto A., Harner C., Fu F.H. (2001). A pilot study on the relationship between physical impairment and activity restriction in persons with anterior cruciate ligament reconstruction at long-term follow-up. Knee Surg. Sports Traumatol. Arthrosc..

[34] Slagers A.J., van den Akker-Scheek I., Geertzen J.H.B., Zwerver J., Reininga I.H.F. (2019). Responsiveness of the anterior cruciate ligament-Return to Sports after Injury (ACL-RSI) and Injury - Psychological Readiness to Return to Sport (I-PRRS) scales. J. Sports Sci..

[35] Webster K.E., Nagelli C.V., Hewett T.E., Feller J.A. (2018). Factors Associated With Psychological Readiness to Return to Sport After Anterior Cruciate Ligament Reconstruction Surgery. Am. J. Sports Med..

[36] Webster K.E., Feller J.A. (2021). Evaluation of the Responsiveness of the Anterior Cruciate Ligament Return to Sport After Injury (ACL-RSI) Scale. Orthop. J. Sports Med..

[37] Akhtar M.A., Bhattacharya R., Keating J.F. (2016). Generalised ligamentous laxity and revision ACL surgery: Is there a relation?. Knee.

[38] Vaishya R., Hasija R. (2013). Joint hypermobility and anterior cruciate ligament injury. J. Orthop. Surg..

[39] Sundemo D., Senorski E.H., Samuelsson K. (2021). Editorial Commentary: Diagnosis and Treatment of Generalized Joint Hypermobility in Patients With Anterior Cruciate Ligament Injury. Arthroscopy.

[40] Zsidai B., Kaarre J., Svantesson E., Piussi R., Musahl V., Samuelsson K., Senorski E.H. (2024). The days of generalised joint hypermobility assessment in all patients with ACL injury are here. Br. J. Sports Med..

[41] Choi N.H. (2024). Editorial Commentary: Increased Lateral Femoral Condyle Ratio, Increased Posterior Tibial Slope, and Narrow Notch Width Are all Risk Factors for Anterior Cruciate Ligament Tear, and Anterior Cruciate Ligament Graft Tear. Arthroscopy.

[42] Wang S., Ma J., Tian C., Feng Z., Xiang D., Tang Y., Geng B., Xia Y. (2024). Decreased sagittal slope of the medial tibial spine and deep concavity of the lateral tibial spine are risk factors for noncontact anterior cruciate ligament injury. Knee Surg. Sports Traumatol. Arthrosc..

[43] Korthaus A., Krause M., Pagenstert G., Warncke M., Brembach F., Frosch K.-H., Kolb J.P. (2022). Tibial slope in the posterolateral quadrant with and without ACL injury. Arch. Orthop. Trauma Surg..

[44] Ravi V., Rehman M., Xia S., Chhabra A., Silva F.D. (2025). Association of tibial slope alterations with anterior cruciate ligament (ACL) injury and mucoid degeneration. Skelet. Radiol..

[45] TALK: Toolkit for Adolescent Care. University of Minnesota Center for Healthy Youth Development. https://chyd.umn.edu/talk-toolkit-adolescent-care.

[46] National Academies of Sciences, Engineering, and Medicine (2019). The Promise of Adolescence: Realizing Opportunity for All Youth.

[47] Cunha J.P. Health Topics: What Are the Three Phases of Adolescence? 2025. https://www.emedicinehealth.com/what_are_the_three_stages_of_adolescence/article_em.htm.

[48] Grodman L.H., Beaulieu M.L., Ashton-Miller J.A., Wojtys E.M. (2023). Levels of ACL-straining activities increased in the six months prior to non-contact ACL injury in a retrospective survey: Evidence consistent with ACL fatigue failure. Front. Physiol..

[49] Nyland J., Pyle B. (2022). Self-Identity and Adolescent Return to Sports Post-ACL Injury and Rehabilitation: Will Anyone Listen?. Arthrosc. Sports Med. Rehabil..

[50] Nyland J., Pyle B., Krupp R., Kittle G., Richards J., Brey J. (2022). ACL microtrauma: Healing through nutrition, modified sports training, and increased recovery time. J. Exp. Orthop..

[51] Brewer B.W., Cornelius A.E. (2010). Self-Protective Changes in Athletic Identity Following Anterior Cruciate Ligament Reconstruction. Psychol. Sport Exerc..

[52] Brewer B.W., Chatterton H.A. (2024). Athletic Identity and Sport Injury Processes and Outcomes in Young Athletes: A Supplemental Narrative Review. J. Funct. Morphol. Kinesiol..

[53] Marmura H., Tremblay P.F., Getgood A.M.J., Bryant D.M. (2022). The Knee Injury and Osteoarthritis Outcome Score Does Not Have Adequate Structural Validity for Use With Young, Active Patients With ACL Tears. Clin. Orthop. Relat. Res..

[54] Brewer B.W., Van Raalte J.L., Linder D.E. (1993). Athletic identity: Hercules’ muscles or Achilles heel?. Int. J. Sport Psychol..

[55] Brewer B.W., Cornelius A.E. (2001). Norms and Factorial Invariance of the Athletic Identity Measurement Scale (AIMS). Acad. Athl. J..

[56] Myer G.D., Jayanthi N., Difiori J.P., Faigenbaum A.D., Kiefer A.W., Logerstedt D., Micheli L.J. (2015). Sport Specialization, Part I: Does Early Sports Specialization Increase Negative Outcomes and Reduce the Opportunity for Success in Young Athletes?. Sports Health.

